# Threshold Dose of Three Types of Quantum Dots (QDs) Induces Oxidative Stress Triggers DNA Damage and Apoptosis in Mouse Fibroblast L929 Cells

**DOI:** 10.3390/ijerph121013435

**Published:** 2015-10-26

**Authors:** Ting Zhang, Yiqing Wang, Lu Kong, Yuying Xue, Meng Tang

**Affiliations:** 1 Key Laboratory of Environmental Medicine Engineering, Ministry of Education, School of Public Health, Southeast University, Nanjing 210009, China; E-Mails: zhangting@seu.edu.cn (T.Z.); wyq336@126.com (Y.W.); konglu@seu.edu.cn (L.K.); yyxue@seu.edu.cn (Y.X.); 2 Jiangsu Key Laboratory for Biomaterials and Devices, Southeast University, Nanjing 210009, China; 3 Wuxi Center for Disease Control and Prevention, Wuxi 214023, China

**Keywords:** oxidative stress, apoptosis, DNA damage, threshold doses, cytotoxicity

## Abstract

Although it has been reported that fluorescent quantum dots (QDs) have obvious acute toxic effects *in vitro*, their toxic effects at low doses or threshold doses are still unknown. Therefore, we evaluated the biological histocompatibility and *in vitro* toxicity of three types of QDs at threshold doses. Also, we compared the toxic effects of QDs with different raw chemical compositions and sizes. The results showed that low concentrations of QDs (≤7 μg/mL) had no obvious effect on cell viability and cell membrane damage, oxidative damage, cell apoptosis or DNA damage. However, QD exposure led to a significant cytotoxicity at higher doses (≥14 μg/mL) and induced abnormal cellular morphology. In addition, when comparing the three types of QDs, 2.2 nm CdTe QDs exposure showed a significantly increased proportion of apoptotic cells and significant DNA damage, suggesting that size and composition contribute to the toxic effects of QDs. Based on these discussions, it was concluded that the concentration (7 μg/mL) may serve as a threshold level for these three types of QDs only in L929 fibroblasts, whereas high concentrations (above 14 μg/mL) may be toxic, resulting in inhibition of proliferation, induction of apoptosis and DNA damage in L929 fibroblasts.

## 1. Introduction

Quantum dots (QDs), fluorescent semiconductor nanocrystals, are a very interesting class of nanomaterials with important imaging applications in biology and medicine [1], such as molecular imaging [2,3], virus trafficking study [4], live cell labeling and tracking [5], cancer targeting and diagnosis [6], cancer detection [7], drug delivery [8], *etc.* However, it was realized in their development that the use of these materials poses serious concerns about toxicity and safety, especially because the most popular and well-studied QDs contain cadmium: CdSe, CdTe, and CdS [9,10].

Recently, a number of *in vitro* studies have focused on understanding the potential toxic effects of QDs. The potential adverse effects of QDs have been reported to be closely associated with their core [11,12,13]. The toxicity of uncoated core CdSe or CdTe-QD has been discussed in several reports and is associated, in part, with free cadmium present in the particle suspensions or released from the particle core intracellularly [14,15]. Lu *et al.* [14] found that CdTe-QDs were cytotoxic in HepG2 cells at 100 nmolar concentration and increased gene expression (MT1A and CYP1A1). The cytotoxicity observed in these studies was found to be consistent with cadmium toxicity from the QD core. Bhatia *et al.* [15] showed that surface oxidation of QDs led to the formation of reduced Cd on the QD surface and release of free cadmium ions, which correlated with cell death. Therefore, cadmium toxicity from QD cores is likely to be a significant contribution to QD toxicity.

As with pharmacological studies, toxicity studies face the same difficulties in terms of size, dose, and exposure—Underscoring the need for rigorous physicochemical characterization of QDs. Particle size is critical to the biological actions of nanoparticles [16]. For QDs, several studies have reported that there is an inverse relationship between quantum dot size or concentration and their adverse effects, smaller sizes and higher concentrations being more cytotoxic. Zhang *et al.* [17] found that CdTe nanoparticles elicited cytotoxicity in a concentration- and size-dependent manner, with smaller-sized particles exhibiting somewhat higher potency. Lovric *et al.* [18,19] showed 2.2 nm CdTe-QD had greater toxicity as compared to larger 5.2 nm particles. Additionally, smaller particles were found localizing in and around the nucleus of the cell, while larger 5.2 nm particles were distributed within the cytoplasm. In addition to size, the dose is also another key factor determining the QDs toxicity. The relationships between the dose or concentration and toxicity have been discussed in several reports [20,21]. Munari *et al.* [20] found that CdS QDs were highly cytotoxic at high concentrations (10 and 50 μg/mL), and exhibited a concentration-dependent genotoxicity in the sub-toxic range (0.01–1 μg/mL) after 24 h exposure. Song *et al.* [21] found that CdTe QDs were cytotoxic in variety of cell lines (HeLa, MCF-7, NIH/3T3 cells) in a dose-dependent manner and inhibited their growth including the decrease of cell metabolic activity, the shrinkage of cells, the breakage of chromatin, the damage of cell membrane integrity, and the fragmentation of mitochondria. Although these toxicological studies have shown the toxicity results of cell death for some primary or immortalized cell lines, the understanding of the relationship between the exposure dose and physicochemical characteristics of QDs governed cytotoxicity (*i.e.*, genotoxicity) remains limited.

In this study, we synthesized CdTe/CdSe QDs and investigated the relationship among particle size, dose, and *in vitro* effects of different types of QDs, especially focusing on apoptosis and DNA damage, using L929 mouse fibroblast.

## 2. Experimental Section

### 2.1. Preparation and Characterization of CdTe/CdSe QDs

The three types of CdTe/CdSe QDs, used in this study were 2.2 and 3.5 nm in size and synthesized by the Department of Biomedical Engineering, Southeast University, China. More details are available in a previously published report [22,23,24]. The transmission electron microscopy (TEM) image was taken by a JEM 2100 microscope (JEOL, Tokyo, Japan) with an acceleration voltage of 200 kV. The particle size and zeta potential of the QDs in pure water and DMEM/12 medium were measured with a Malvern Zetasizer (Nano-ZS, Malvern Instruments, Worcestershire, UK).

### 2.2. Hemolysis Test

The hemolytic activity of the polymers was investigated according to a previously published report [25,26]. Ethylenediamine tetraacetic acid (EDTA) (NanJing SunShine Biotechnology Co., LTD. Nanjing, China)-stabilized human blood samples were freshly obtained from hospital of southeast university. First, 5 mL of blood sample was added to 10 mL of phosphate buffered saline (PBS) (NanJing SunShine Biotechnology Co., LTD. Nanjing, China), and then red blood cells (RBCs) were isolated from serum by centrifugation at 1000 rpm for 10 min. The RBCs were further washed five times with 10 mL of PBS solution. The purified blood was diluted to 50 mL of PBS. Prior to QDs exposure, the absorbance spectrum of the positive control supernatant was checked and used only if it was in the range of 0.50–0.55 optical density units to reduce sample difference from different donors. Herein, RBC incubation with distilled (D.I.) water and PBS were used as the positive and negative controls, respectively. Then 0.5 mL of diluted RBC suspension was added to 2 mL of CdTe/CdSe QDs solutions at systematically varied concentrations and mixed by vortexing. The CdTe/CdSe QDs suspended in PBS solutions with different concentrations (10, 20, 40, 80, and 160 μg/mL) were prepared immediately before red blood cell incubation by serial dilution. All the sample tubes were kept in static condition at room temperature for 30 min. Finally, the mixtures were centrifuged at 3000 rpm for 10 min, and 100 μL of supernatant of all samples was transferred to a 96-well plate. The absorbance values of the supernatants at 540 nm were determined by using a microplate reader with absorbance at 655 nm as a reference. The percent hemolysis of RBCs was calculated using the following formula: percent hemolysis = ((sample absorbance—Negative control absorbance)/(positive control absorbance—Negative control absorbance)) × 100.

### 2.3. Cell Culture and Treatments

Mouse fibroblast L929 fibroblasts were purchased from Shanghai Institute of Cell Biology, Chinese Academy Sciences. L929 fibroblasts were one of the first and most widely used cells in cytotoxicity test. L929 fibroblasts used in the cytotoxicity tests were maintained in DMEM/12 medium supplemented with 10% FBS (NanJing SunShine Biotechnology Co., LTD. Nanjing, China), penicillin 100 U/mL (Gibco, Carlsbad, CA, USA), and streptomycin 100 μg/mL (Gibco, Carlsbad, CA, USA), and cultured at 37 °C in a 5% CO_2_ humidified incubator. Cells in the logarithmic growth phase were used in all the experiments. The test suspension of CdTe/CdSe QDs was prepared using the culture media and dispersed for 10 min by using a sonicator (Branson Inc., Danbury, CT, USA) to prevent aggregation. The cells were treated with various concentrations of particles according to the time schedule which is designated in the following section of each toxicological study. All measurements were conducted in duplicate in three independent experiments.

### 2.4. Viability Analysis

Cell viability was measured using the (3-[4,5-dimethylthiazol-2-yl]-2,5-diphenyltetrazolium bromide) (MTT) colorimetric assay. L929 fibroblasts were grown until they reached 80% confluence, then seeded into 96-well culture plates at a density of 8000 cells/well in a total volume of 200 µL and allowed to attach and grow for 24 h. The supernatant in each well was then replaced with DMM F/12 medium containing 0, 5.85, 8.78, 13.17, 19.75, 29.63, 44.44, 66.66, 100, and 150 μg/mL concentrations of CdTe/CdSe QD. After 24 h of incubation, 100 µL of MTT (Sigma-Aldrich, Shanghai, China) was added to each well. After 4 h incubation, the supernatant was removed and 150 µL dimethylsulfoxide (DMSO, Sigma-Aldrich, Shanghai, China) was added to each well. Samples were then shaken for 15 min at 37 °C to solubilization formazan products. Absorbance was quantified at a wavelength of 490 nm using a POLARstar OPTIMA microplate reader (Mithras LB940, Berthold, Germany). Cell viability was expressed as the ratio between the amounts of formazan from cells of the non-treated control group. All experiments were performed in triplicate.

### 2.5. Microscopic Observations

After incubation with CdTe/CdSe QDs, changes in morphology and detachment of L929 fibroblasts from the dish were observed using a Nikon inverse phase contrast microscope (Nikon TMS, Nikon, Japan) equipped with an objective (Plan 10/0.30 DL/Ph1, Nikon, Japan) of ×100 magnification.

### 2.6. LDH Release Assay

Lactate dehydrogenase (LDH) is released from L929 fibroblasts exposed to three different CdTe/CdSe QDs. The LDH activity of the supernatant of the culture medium was determined using a commercial LDH cytotoxicity test (Nanjing Jiancheng Chemical Industrial Co.Ltd, Nanjing, China) according to the manufacturer’s instructions. In brief, 5 × 10^4^ cells were seeded into each well of a 96-well plate. After 24 h incubation, cells were treated with three different CdTe/CdSe QDs and four final concentrations (0, 3.5, 7, and 14 μg/mL). After a further 24 h incubation period, 50 μL of medium overlying cells was used for LDH analysis. Absorption of light at 560 nm was measured using a spectrophotometer. All experiments were performed in triplicate. 

### 2.7. Oxidative Damage

In addition to the analysis of cytotoxicity and LDH release, four oxidative stress markers, including hydroxyl radical (•OH), malondialdehyde (MDA), superoxide dismutase (SOD) and glutathione peroxidase (GSH-Px) were evaluated. After L929 fibroblasts exposure to different concentrations (3.5, 7, and 14 μg/mL) of CdTe/CdSe QD for 24 h, washed once with ice-cold PBS, and lysed in ice-cold RIPA lysis buffer containing 1 mM phenylmethylsulphonyl fluoride (PMSF) (Beyotime, Shanghai, China) for 30 min. After centrifuging the lysates at 12,000 rpm, 4 °C for 10 min, the supernatants were collected for measurements of the production of •OH and MDA, the activities of SOD and GSH-Px. All examinations were carried out using commercially available kits (Nanjing Jiancheng Chemical Industrial Co. Ltd, Nanjing, China) according to the manufacturer’s instructions. The protein concentrations of these extracts were determined by performing the bicinchoninic acid (BCA) protein assay (Beyotime, Shanghai, China).

### 2.8. Annexin V-FITC/Propidium Iodide Apoptosis Assay

Normal, apoptotic, and necrotic cells were distinguished using an Annexin V-FITC/propidium iodide assay kit (KeyGEN Biotech, Nanjing, China) according to the manufacturer’s instructions. L929 fibroblasts were plated in a six-well culture plate at a density of 1.5 × 10^5^ cells/mL with 2 mL/hole for 12 h. Then we discarded the medium, and the cells were washed with PBS then treated with QDs (0, 3.5, 7, 14 µg/mL) for 24 h. Thereafter, cells were harvested and washed with PBS, resuspended in 400 µL of binding buffer to a density of 1 × 10^6^ cells/mL, and 5 µL of Annexin V-FITC was then added to the samples. After incubation for 15 min at 4 °C in the dark, 10 µL of propidium iodide was added and the cells were incubated for 5 min. Flow cytometry (BD FACSCalibur, San Jose, CA, USA) analysis was performed within 15 min.

### 2.9. Comet Assay

The comet assay (single cell gel electrophoresis) was used to investigate DNA damage in terms of strand breaks (SBs) and alkali-labile sites (ALS). To assess oxidatively damaged DNA, an additional step of Formamido Pyrimidine DNA Glycosylase (FPG) treatment was performed when L929 fibroblasts were cultured in six-well plates with exposure to CdTe/CdSe QDs for 2 h. The comet assay was then performed as previously described [26]. The slides were coded, and one scorer performed the comet analysis using a fluorescence microscope (Olympus, Tokyo, Japan). At least 90 cells per sample (three replicates, each with 30 cells/slide) were analyzed using the comet image analysis software (Youworks, Tokyo, Japan), and the percentage of DNA, tail length, and tail moment in the comet tail were used as a measure of the amount of DNA damage. We used L929 fibroblasts exposed to 2.5 µL of 30% hydrogen peroxide as a positive control, and untreated L929 fibroblasts as a negative control.

### 2.10. Statistical Analysis

All data was expressed as mean ± standard deviation. Results were compared by one-way analysis of variance (ANOVA) followed by Dunnett’s test for comparison of treatment groups to the negative control group and LSD test for pairwise comparisons among treatment groups. A value of *p* < 0.05 was considered to be statistically significant.

## 3. Results

### 3.1. Synthesis and Characterization of CdTe/CdSe QDs

**Figure 1:**
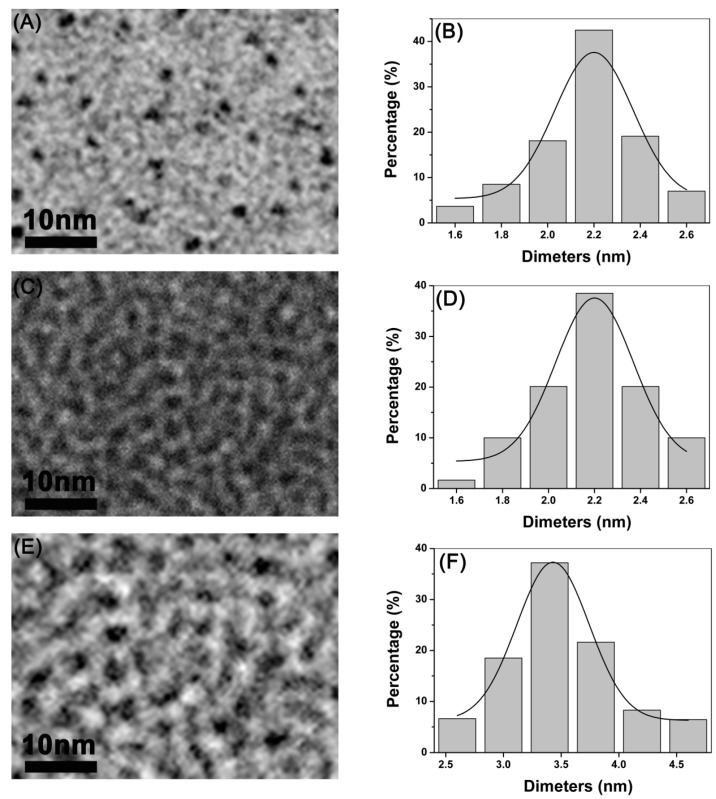
Characterization of QDs. (**A**), (**C**) and (**E**) represents the TEM characterization of 2.2 nm CdSe QDs, 2.2 nm CdTe QDs and 3.5 nm CdTe QDs respectively; (**B**), (**D**) and (**F**) depict size distribution by DLS characterization of 2.2 nm CdSe QDs, 2.2 nm CdTe QDs and 3.5 nm CdTe QDs, respectively.

The microstructural characterization of the CdTe/CdSe QDs investigated in this study is summarized in Figure 1. The TEM assessment showed the shape and morphology of the CdTe/CdSe QDs used in this study (Figure 1A,C,E). The analysis based on the nanoparticle size analyzer suggested that the diameter of CdTe/CdSe QDs was about 2.2 nm and 3.5 nm.

### 3.2. Determination of the Hemolytic Activity

**Figure 2:**
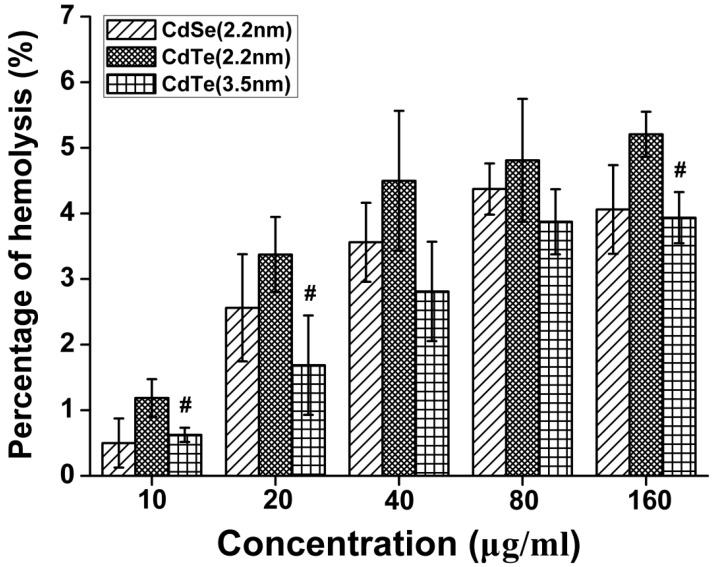
Percentage of hemolysis of RBCs incubated with three types of QDs at different concentrations ranging from 10 to 160 μg/mL for 30 min. Data are the mean ± SD of three separate experiments. Significance was indicated by: ^#^ *p* < 0.05 *vs.* 2.2 m CdTe QDs.

The biological histocompatibilities of the CdTe/CdSe QDs were studied via hemolysis experiments. The release of hemoglobin was used to quantify the membrane-damaging properties of the different size of CdTe/CdSe QDs. Erythrocytes were incubated with five different QDs concentrations in the range of 10~160 μg/mL for 30 min. As shown in the Figure 2, the hemolysis percentage of RBCs increases in a concentration-dependent manner in three types of QDs. When exposed to the dose up to 80 μg/mL, the hemolysis of three types of QDs was slightly increased, and the highest of the hemolysis percentage was 2.2 nm CdTe (5.12%). Under these conditions, 2.2 nm CdSe QDs, 2.2 nm CdTe and 3.5 nm CdTe QDs showed no hemolytic effects up to 160 μg/mL, indicating no detectable disturbance of the red blood cell membranes. However, it is apparent that the hemolysis of 2.2 nm CdTe (5.21%) was significantly different compared with the hemolysis of 3.5 nm CdTe QDs (3.94%), and the difference was significant (*p* < 0.05). This result demonstrates that the smaller particles have higher hemolytic activity than the larger particles.

### 3.3. Concentration- and Size-Dependent Cytotoxicity of QDs Induced in L929 Mouse Fibroblasts

**Figure 3:**
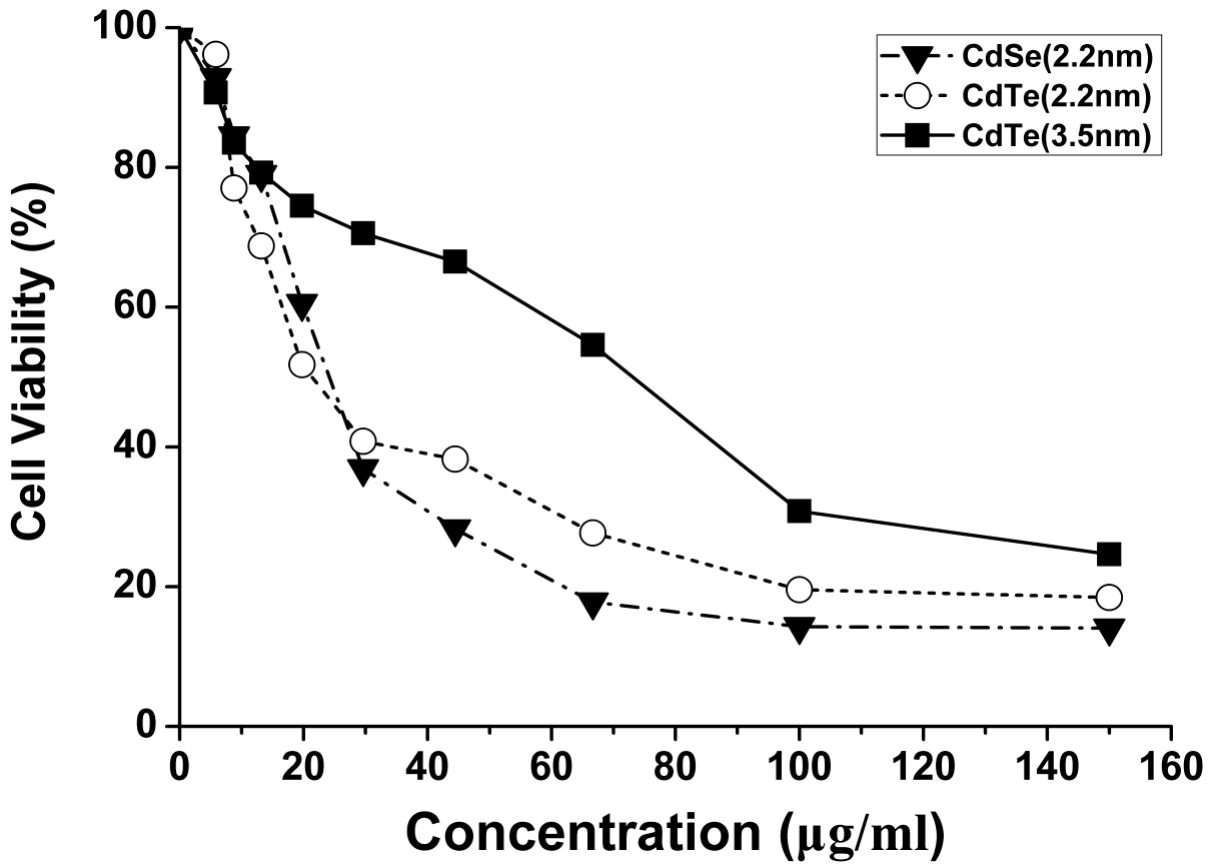
The viability of L929 mouse fibroblasts after a 24 h exposure to three types of QDs (5.85–150 μg/mL) was assessed using MTT assay. The results are expressed as % mitochondrial activity.

To evaluate the possible toxicity of QDs on L929 fibroblasts, cell viability was determined after exposing to different sizes of CdTe/CdSe QDs for 24 h. As indicated in Figure 3, viability of L929 fibroblasts induced by CdTe/CdSe QDs showed no significant change at the concentration of 0~8.78 μg/mL between three types of QDs. As the exposure concentration increased, CdTe/CdSe QDs inhibited cell growth remarkably on L929 fibroblasts in 19.75 μg/mL treated group at 24 h. Up to 19.75~150 μg/mL, the cell viability rate of 2.2 nm CdTe QDs and 2.2 nm CdSe QDs treated group decreased greatly, which was lower than that of 3.5 nm CdTe QDs treated group. Overall, the cell viability rate as shown by Figure 3 was decreased by treatment with QDs both size- and concentration-dependently. Concentrations for which the cell viability was inhibited by 20% and 50% (IC_20_ and IC_50_) were determined by the method of Bliss [27]. The calculated IC_20_ and IC_50_ value of three types of QDs was summarized in Table 1. The IC-values were further used for analysis of cell death, mitochondrial damage, DNA damage and intracellular ROS formation.

**Table 1:** The calculated IC_20_ and IC_50_ value of three types of QDs (μg/mL).

QDs	IC_20_	IC_50_
2.2 nm CdSe QDs	10.84	27.16
2.2 nm CdTe QDs	7.38	25.83
3.5 nm CdTe QDs	15.53	59.82

### 3.4. Change of Cellular Morphology

**Figure 4:**
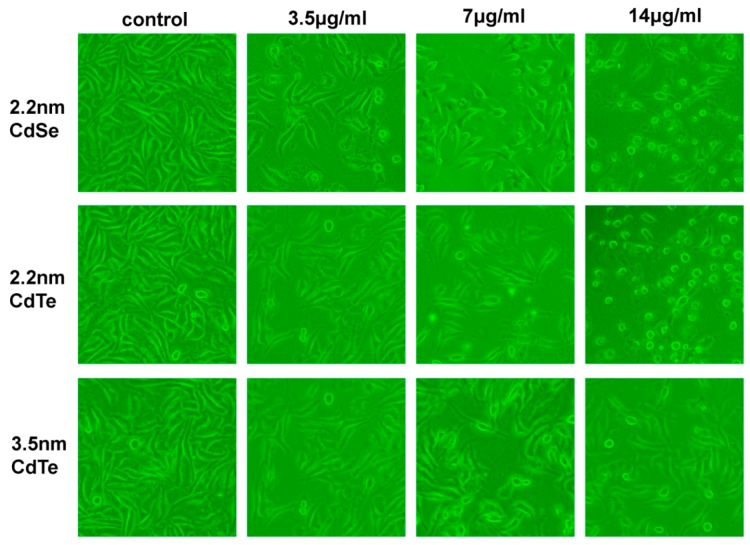
Three types of QDs induced morphological changes. L929 mouse fibroblasts were cultured without (control) and with 3.5, 7, and 14 μg/mL of QDs and analyzed after 24 h of exposure with microscopy. The photographs shown correspond to a representative experiment of three independent assays.

Change of cellular morphology was directly related with cell viability. The morphological changes of L929 fibroblasts became more and more obvious (Figure 4) with the increased dosages (3.5, 7, and 14 μg/mL). Usually, untreated L929 fibroblasts are large, spindle-shaped, adherent cells growing as a confluent monolayer. After exposed to QDs, cell density reduction, irregular shape and cellular shrinkage were observed. Higher concentrations of QDs generated more pronounced cell debris and changes in morphology, such as cell lysis, loss of spindle shape and sometimes detachment from the bottom. Compared with control group, the cell density in the 14 μg/mL treated group was obviously reduced after 24 h exposure.

### 3.5. QDs Caused Membrane Damage in L929 Mouse Fibroblasts Detected by LDH Assay

**Figure 5:**
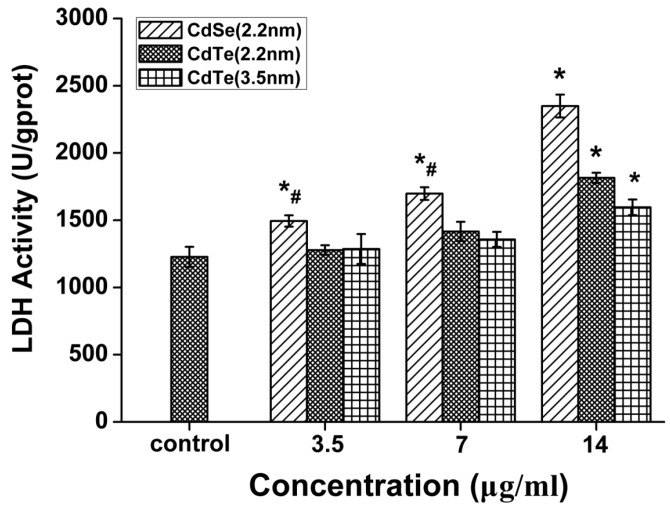
LDH leakage from L929 mouse fibroblasts after 24 h treatment with three types of QDs (3.5, 7, and 14 μg/mL) at 24 h. Data are the mean ± SD of three separate experiments. Significance was indicated by: * *p* < 0.05 *vs.* control cells. ^#^ *p* < 0.05 *vs.* 2.2 m CdTe QDs.

To assess the damage to cell membranes, as the initial point of interaction with QDs, the extracellular concentration of LDH was quantified after exposure to three different concentrations (3.5, 7 and 14 μg/mL) for 24 h (Figure 5). Compared with the controls, LDH levels in cell medium were gradually elevated as QDs concentrations increased. Following exposure to 2.2 nm CdSe QDs, 2.2 nm CdTe and 3.5 nm CdTe QDs at the highest dosage levels, LDH releases were increased by 191.4%, 147.9% and 130.1%, respectively, significantly higher than the untreated control (*p* < 0.05). However, the effect was not as significant as that on cellular viability. In addition, it was noted that statistically significant difference was found when comparing the effects between 2.2 nm CdSe QDs and 2.2 nm CdTe QDs at the same dosage level.

### 3.6. Oxidative Stress-Dependent Toxicity of CdTe/CdSe QDs

**Figure 6:**
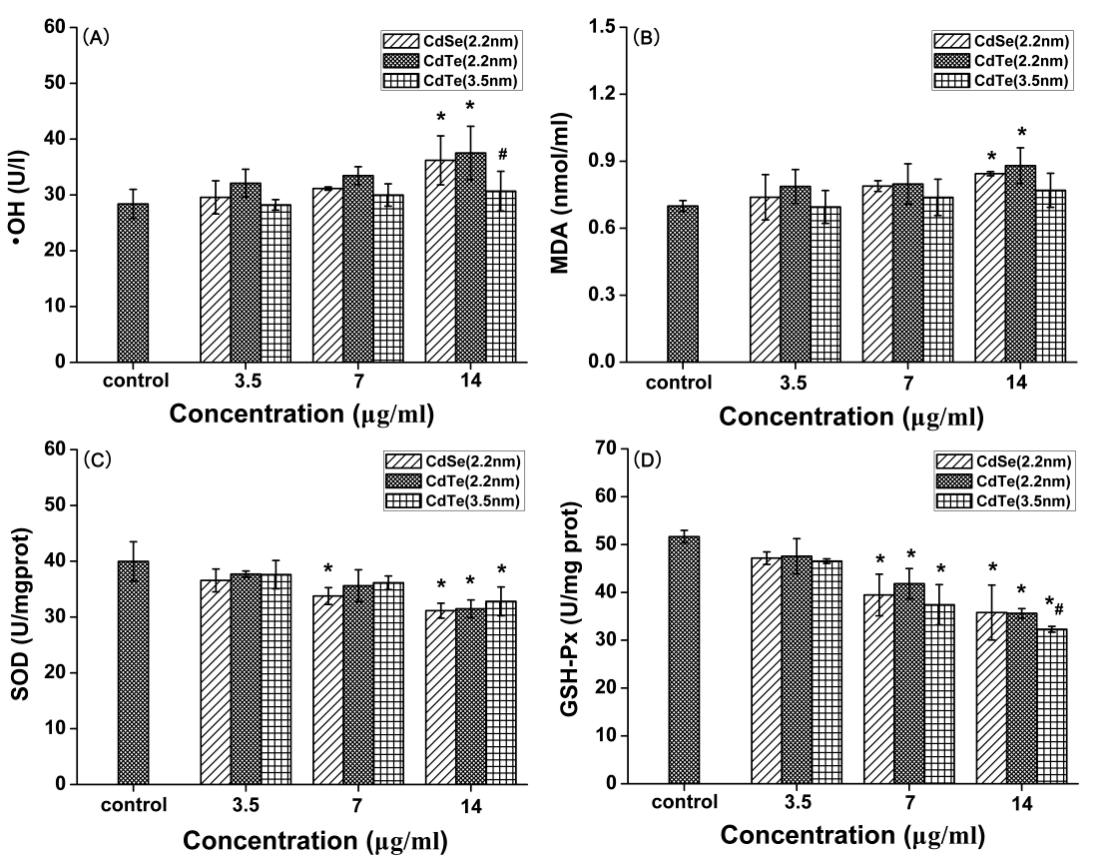
The hydroxyl radical (•OH), malondialdehyde (MDA) levels, superoxide dismutase (SOD) and glutathione peroxidase (GSH-Px) activities in L929 mouse fibroblasts after being treated with three types of CdTe QDs (3.5, 7, and 14 μg/mL) at 24 h. Data are the mean ± SD of three separate experiments. Significance was indicated by: * *p* < 0.05 *vs.* control cells. ^#^ *p* < 0.05 *vs.* 2.2 m CdTe QDs.

Oxidative stress is an important mechanism of cytotoxicity with respect to nanoparticle exposure. Four oxidative stress markers, including hydroxyl radical (•OH), malondialdehyde (MDA), superoxide dismutase (SOD) and glutathione peroxidase (GSH-Px) were evaluated in L929 fibroblasts treated with different sizes of CdTe/CdSe QDs for 24 h. The •OH radical is the most reactive ROS and triggers extensive cellular damage. The increased •OH generation was observed in a concentration dependent manner following the exposure of 3.5, 7, and 14 μg/mL of the three types of QDs. After treatment with 14 μg/mL of QDs for 24 h, the •OH production in L929 fibroblasts was found to be significantly increased compared with control groups (*p* <0.05) (Figure 6A).

In order to elucidate the lipid peroxidation induced by QDs, the MDA content was measured. Each type of QD elevated the intracellular MDA content in a concentration-dependent manner (Figure 6B). 2.2 nm CdSe QDs and 2.2 nm CdTe at 14 μg/mL for 24 h resulted in the greatest MDA generation and increased significantly compared with control groups (*p* < 0.05). Compared with the •OH measurement, parallel results were observed in this experiment. For example, the greatest increase of the MDA content also occurred in 2.2 nm CdTe QDs exposed cells. Furthermore, the MDA content of 2.2 nm CdTe more significantly increased than that of 3.5 nm CdTe QDs.

SOD and GSH-Px are the main antioxidant enzymes in cells, and activities of these enzymes reflect the enzymatic antioxidant capacity of cells. As presented in Figure 6C,D, the activities of SOD and GSH-Px were decreased in a concentration-dependent manner. The activities of SOD were significantly lower in three types of QD-exposed L929 fibroblasts at a concentration of 14 μg/mL in comparison with the control (*p* < 0.05), and effect on the activities of GSH-Px were found at 7 to 14 μg/mL (*p* < 0.05). We also found that the SOD activities were highly correlated with MDA levels in all the three particle groups.

Overall, three types of QDs resulted in concentration-dependent reduction of SOD and GSH-Px activities and generation of •OH and MDA content, which reflected the oxidative stress in the L929 fibroblasts. However, the comparative analysis of these oxidative effects demonstrated that there are significant differences between the three types. Concretely, 2.2 nm CdTe QDs nanoparticles induced the most significant oxidative stress while 3.5 nm CdTe induced the least. In addition, the oxidative stress of 2.2 nm CdTe QDs appeared slightly more effective than that of 2.2 nm CdSe QDs.

### 3.7. CdTe/CdSe QDs Induced Apoptosis

**Figure 7:**
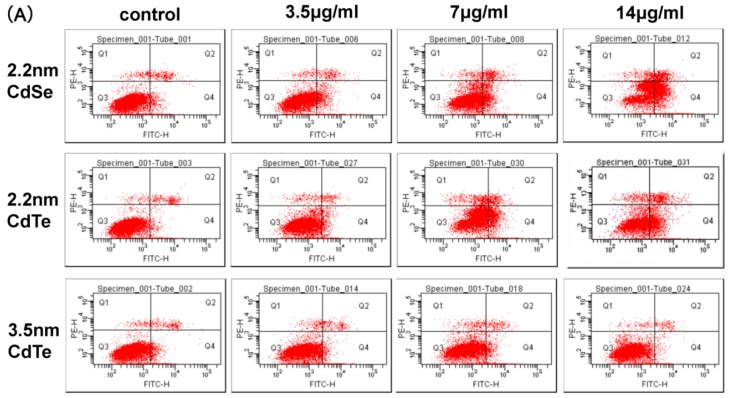

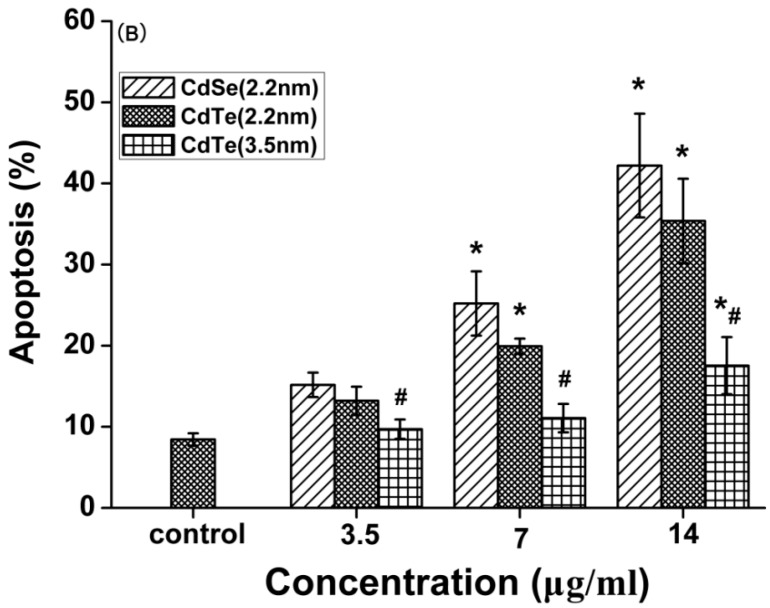
The analysis of the apoptosis in the cells. (**A**) Flow cytometric evaluation of the effect of three types of QDs on L929 mouse fibroblasts apoptosis and necrosis after 24 h of treatment with 3.5, 7, and 14 μg/mL CdTe QDs. Cells were stained using an apoptotic detection kit (PI/annexin V-FITC). Quadrant (Q)1 shows the percentage of necrotic cells (PI positive cells), Q2 shows the percentage of cells of late apoptotic phase (Double positive cells), Q3 shows the percentage of normal viable cells and Q4 shows the percentage of early apoptotic cells (annexin V-FITC positive cells); (**B**) shows the percentage of apoptosis cell in the early and late stages (Q2+Q4). Data are the mean ± SD of three separate experiments. Significance was indicated by: * *p* < 0.05 *vs.* control cells. ^#^ *p* < 0.05 *vs.* 2.2 m CdTe QDs.

To quantitatively evaluate whether the effect of QDs on cell viability was associated with the induction of apoptosis, surface exposure of phosphatidylserine (PS)which is an early event of apoptotic program was assessed by double staining with Annexin V-FITC-conjugated and propidium iodide (PI) by flow cytometry analysis (Figure 7A). All QDs affected the percentage of apoptotic cells in a concentration-dependent manner when they were added in the concentration range 0–14 μg/mL for up to 24 h to the cells (Figure 7B). When exposed to a concentration up to 7 μg/mL, the percentage of apoptotic cells of three types of QDs was increased, which was significantly higher than that of the control. Furthermore, it is apparent that the percentage of apoptotic cells of 2.2 nm CdTe (35.37%) was significantly increased compared with the percentage of apoptotic cells of 3.5 nm CdTe QDs (17.53%), and the difference was significant (*p* < 0.05). These results showed that QDs induced size- and concentration- dependent apoptotic cell death.

### 3.8. DNA Damage of L929 Mouse Fibroblasts Induced by QDs

The comet assay is already recognized as the most sensitive method available for measuring DNA damage. In this assay, there were significantly increased in tail length, percentage of DNA in tail, tail moment and olive tail moment after L929 fibroblasts were treated with three types of QDs at the examined concentrations (Table 2). Our data indicated that the DNA damage caused by QDs was getting more serious with the concentration increasing; however, there was no significant difference between them at the 7 μg/mL dosage level. When exposed to a dose of up to 14 μg/mL, the genotoxicity of the same size of QDs was not obvious; however, the genotoxicity of different sizes of CdTe was significant, and 2.2 nm CdTe caused more DNA damage than 3.5 nm CdTe QDs.

**Table 2:** Changes in the levels of DNA damage (tail length, % DNA in tail, tail moment and olive tail moment) in L929 mouse fibroblasts.

Group	Dose (μg/mL)	Tailing Rate (%) (*n* = 200)	Tailing Cell (*n* = 20)		
			DNA in Tail (%)	Tail Length	Olive Tail Moment
negative control	—	11.34 ± 2.17	4.67 ± 4.23	32.20 ± 20.18	7.27 ± 6.23
positive control	—	88.25 ± 5.45 ^a^	24.83 ± 20.17 ^a^	207.45 ± 84.69 ^a^	47.56 ± 23.66 ^a^
2.2 nm CdSe	3.5	14.67 ± 3.68	6.33 ± 5.02	37.64 ± 25.42	9.16 ± 7.30
	7	17.24 ± 5.58	13.53 ± 10.46	89.27 ± 40.15	20.43 ± 18.33
	14	25.55 ± 5.84 ^a^	24.00 ± 19.68 ^a^	138.35 ± 72.64 ^a^	28.78 ± 20.73 ^a^
2.2 nm CdTe	3.5	13.22 ± 4.78	5.67 ± 4.64	38.86 ± 29.95	11.41 ± 6.27
	7	18.85 ± 5.97	14.00 ± 12.94 ^a^	78.80 ± 45.85 ^a^	22.28 ± 18.69
	14	24.73 ± 4.57 ^a^	26.67 ± 20.74 ^a^	123.55 ± 65.73 ^a^	27.58 ± 16.62 ^a^
3.5 nm CdTe	3.5	13.35 ± 3.67	5.07 ± 4.48	38.37 ± 26.84	9.81 ± 6.16
	7	14.49 ± 4.83 ^a^	12.67 ± 9.43	59.27 ± 30.15	18.62 ± 12.99
	14	19.25 ± 4.14 ^a,b^	23.33 ± 20.27 ^a,b^	94.68 ± 63.86 ^a,b^	20.11 ± 16.30

Values are given as mean ± S.D. of six experiments in each group. ^a^ *p* < 0.05 *vs.* control; ^b^ *p* < 0.05 *vs.* the same dose of 2.2 nm CdTe group.

## 4. Discussion

With the increasing applications of QDs in various commercial products, concerns about their risk to human health and the environment are growing. However, the underlying mechanisms of these adverse effects have not been fully characterized. In this study, different sizes of CdTe QDs and CdSe QDs were produced, carefully characterized and assessed with the same *in vitro* cytotoxicity, genotoxicity, and apoptosis test, as were toxic effect factors.

*In vitro* erythorocyte-induced hemolysis is considered to be a simple and reliable measurement for estimating the blood compatibility of materials which have recently been used for biomedical applications. Also, plasma membrane damage is an important aspect of cellular toxicity in terms of nanoparticle treatment. When cells have plasma membrane damage, the propidium iodide in the solution passively diffuses into the cytoplasm and binds with intracellular DNA or RNA [28]. The behavior of QDs *in vivo* can be predicted by investigating the degree of hemolysis *in vitro* [29]. Hemolysis results showed that a slight hemolysis was produced in all QD groups after 30 min incubation. When the dose was less than 10 μg/mL, all sizes of CdTe QDs and CdSe QDs show a negligible hemolysis (less than 2%) [29]. 2.2 nm CdTe QDs revealed a relatively higher extent of breakdown of the RBCs. The higher hemolytic activity of 2.2 nm CdTe QDs may be due to the smaller QD particle size. The size-dependent cytotoxicity in human RBCs and mammalian cells has also been demonstrated using other types of nanoparticles, such as silica [30,31], graphene oxide [32] and latex [33]. However, the largest observed hemolytic activity was lower than 6% which indicates a wide safety margin in blood-contacting applications and suitability for intravenous administration [34].

To further explore the cytotoxicity of QDs, cell viability as cytotoxicity indicators, were employed to investigate how these QDs interact with L929 fibroblasts. The MTT results of three types of QDs showed concentration-dependent effects on the mitochondrial activity of L929 fibroblasts (Figure 3), indicating that the adherent fibroblast cells were affected by either QDs at any of the employed concentrations. Our data revealed that the QD-induced cytotoxicity increased in a size- and concentration-dependent manner. There was a statistically significant difference between 2.2 nm and 3.5 nm CdTe QDs in mitochondrial activity. The lower viability of 2.2 nm CdTe QDs may be due to the smaller QDs particle size, which influenced the intake and distribution of QDs. Lovrić *et al.* [19] demonstrated QD-induced cytotoxicity was in part dependent on QD size and is characterized by chromatin condensation and membrane blebbing. In the murine microglial N9 cell, line red cationic QDs (5.2 nm) were distributed throughout the cytoplasm. In contrast, green and positively charged QDs (2.2 nm) were often found in the nucleus of N9 cells upon QD exposure. Furthermore, our data also showed that all sizes of QDs induced sharp growth inhibition of L929 fibroblasts when the exposure concentration was above 20 μg/mL. Considering the biological applications of quantum dots, we focused mainly on the cellular effects of low concentrations and investigated the potential molecular mechanism in the current study. From the MTT results, the IC_50_ values of 2.2 nm CdSe QDs, 2.2 nm CdTe QDs and 3.5 nm CdTe QDs, three types of QDs which resulted in low proliferation, were 27.16, 25.83 and 59.82 μg/mL respectively, and when the concentration was 19.75 μg/mL, the same size of CdTe QDs and CdSe QDs showed different cytotoxicity levels. Therefore, these concentrations (3.5, 7, 14 μg/mL) were used in the following experiments.

Exposure to cytotoxic agents can affect cellular morphology, which directly reflects cell injuries [35], cell density reduction, irregular shape as well as cellular shrinkage. With a consequent increase in exposure concentration, cells retracted into a spherical shape and formed clusters in media after detachment from surface. Unlike the hemolysis assay, L929 fibroblasts were grown on the bottom of the assay wells, making factors such as the sedimentation rate, thickness and compactness of QDs aggregates on the top of adherent cells more likely to affect the viability of fibroblasts. LDH release, as an indicator of cell membrane damage, was employed to investigate the degree of injury in L929 fibroblasts. 2.2 nm CdSe QDs revealed a relatively higher extent of breakdown of L929 fibroblasts. This was not in accordance with the results obtained from the MTT assays which showed obvious diversity in cytotoxicity of 2.2 nm CdTe QDs. Therefore, this suggests that QDs can cross the cell membrane and enter the cytoplasm through several different routes, which would change mitochondrial activity rather than injure the cellular membrane [36,37,38].

Oxidative stress has been suggested to play an important role in the mechanism of toxicity of a number of nanoparticles either by the production of ROS or by depleting cellular antioxidant capacity [39,40]. Cellular integrity is affected by oxidative stress when the production of ROS and lipid peroxidation overwhelm the antioxidant defense mechanism [40]. The •OH radical is the most reactive ROS and triggers extensive cellular damage. Oxidative stress is the result of an imbalance in pro-oxidant/antioxidant homeostasis. In the present study, we measured •OH radical production, the concentration of MDA, and antioxidase (SOD and GSH-Px) activity. Our data demonstrate a dose-dependent increase in the formation of •OH and MDA level compared to control, suggesting an increase in lipid peroxidation upon QDs exposure. Conversely, the activities of the antioxidant enzymes SOD and GSH-Px were reduced significantly; these results are in accordance with previous studies [41,42]. Lipid peroxidation is a chain-reaction process that propagates by an intermediate peroxy radical and continues as long as unsaturated lipid molecules in the cell are available [43]. The increase in lipid peroxidation observed in the present study after QD exposure may be due to the increased ROS production leading to membrane damage as indicated by enhanced LDH release. If the level of ROS exceeds the scavenging ability of these antioxidant enzymes, redundant ROS can deplete the activities of antioxidant enzymes and overwhelm the intrinsic antioxidant defenses systems of the cell, which will lead to excess oxidation of lipids, proteins and DNA, and then induce apoptosis [44,45]. Taken together, the results stated above suggest that QDs induced oxidative stress in L929 fibroblasts at a high concentration (≥14 μg/mL), which was probably responsible for the induction of DNA damage and apoptosis.

To further analyze the cell death caused by QDs, apoptosis in L929 fibroblasts were measured (Figure 7). In accordance with LDH results, a significant increase of apoptosis rate was noted at the concentrations (3.5, 7 and 14 μg/mL) of three types of QDs. Rapid-acting metabolic poisons and great physical stress can cause necrosis accompanied by membrane damage. In contrast, apoptosis is a slow-acting form of cell death accompanied by an energy-dependent sequence of events, ultimately resulting in fragmenting nuclei and cytoplasmic organelles; thus, the membrane damage is not a primary event of apoptosis [35,46]. In this study, we found that L929 fibroblast exposure to QDs also caused apoptosis at a concentration 14 μg/mL. Similar results were obtained from our previous studies, which suggested that the AML12 exposured to 2.2 nm CdTe QDs could cause apoptosis [42].

Owing to their small size, some nanoparticles are capable of reaching the nucleus and interacting with DNA [47,48]. They may also exhibit an indirect effect on DNA through their ability to generate ROS [49]. This DNA damage may either lead to carcinogenesis or cell death, thus disrupting normal cell functions. Comet assay is capable of detecting single as well as double DNA strand breaks and alkali labile sites even at low levels of DNA damage [50]. Previous studies have revealed the DNA damaging potential of QDs in somatic cells (human lymphocytes, human lung adenocarcinoma cells, and primary normal human bronchial epithelial cells) and zebrafish [51,52,53,54]. ROSs are known to react with DNA molecules, causing damage to both purine and pyrimidine bases as well as the DNA backbone [55]. The results showed that QDs induced concentration-dependent, highly significant DNA damage which was observed by the induction in the fold change of olive tail moment (OTM), percent tail DNA, and tail length as shown in Table 2. Moreover, the results revealed a similar threshold value for QDs genotoxicity, indicating a severe DNA damage potential in L929 fibroblasts at 7.0 μg/mL. To our knowledge this is the first investigation to prove a threshold concentration of genotoxicity induced by QDs exposure. However, there is a lack of information on possible QDs concentrations in per-implant tissue *in vivo*. Theoretically, genotoxic or even cytotoxic levels may be reached, and further *in vivo* studies are required in order to obtain this very important information.

## 5. Conclusions

In summary, the current study showed that QDs may induce cytotoxic effects in L929 fibroblasts at high exposure concentrations. The QDs were also found to induce oxidative stress which led to DNA damage and subsequent apoptosis in liver cells; however, when the cell was exposed to low concentrations of 3.5 nm and 2.2 nm CdTe QDs (under 7 μg/mL), 3.5 nm and 2.2 nm CdTe QDs (under 7 μg/mL) there was no effect on oxidative stress markers, no obvious cell apoptosis and DNA damage in L929 fibroblasts. From a panoramic view of the QD blood compatibility study, the concentration (7 μg/mL) may serve as a threshold level for these three types of QDs only in L929 fibroblasts. However, whether exposure to low concentrations of QDs could be safe remains uncertain and requires further *in vivo* and other experimental studies.
